# Pelvic vein incompetence and chronic pelvic pain: a case–control study

**DOI:** 10.1111/1471-0528.17485

**Published:** 2023-04-24

**Authors:** Vivak Hansrani, David Riding, Mourad W. Seif, Ann-Louise Caress, Katherine Payne, Jonathan Ghosh, Charles N. McCollum

**Affiliations:** 1 Division of Cardiovascular Sciences, School of Medical Sciences, University of Manchester, Manchester, UK; 2 Manchester Vascular Centre, Manchester University NHS Foundation Trust, Manchester, UK; 3 St Mary's Hospital, Manchester University NHS Foundation Trust, Manchester, UK; 4 Health Services Research, Department of Nursing and Midwifery, School of Human and Health Sciences, University of Huddersfield, Huddersfield, UK; 5 Institute of Population Health, University of Manchester, Manchester, UK

**Keywords:** case–control study, chronic pelvic pain, pelvic venous incompetence, ultrasound, venous

## Abstract

**Objective:**

To investigate the association between chronic pelvic pain (CPP) and pelvic vein incompetence (PVI) or pelvic varices.

**Design:**

Case–control study.

**Setting:**

Gynaecology and vascular surgery services in two teaching hospitals in north-west England.

**Sample:**

A total of 328 premenopausal women (aged 18–54 years), comprising 164 women with CPP and 164 matched controls with no history of CPP.

**Methods:**

Symptom and quality-of-life questionnaires and transvaginal duplex ultrasound for PVI and pelvic varices.

**Main outcome measures:**

Venous reflux of >0.7 s in the ovarian or internal iliac veins (primary outcome) and presence of pelvic varices (secondary outcome). Statistical analysis compared the prevalence of PVI between women with and without CPP using the two-sided chi-square test. Logistic regression was used to compare the odds of having PVI and pelvic varices between women with and without CPP.

**Results:**

Pelvic vein incompetence was found on transvaginal duplex ultrasound in 101/162 (62%) women with CPP, compared with 30/164 (19%) asymptomatic controls (OR 6.79, 95% CI 4.11–11.47, *p* < 0.001). Forty-three of 164 (27%) women with CPP had pelvic varices compared with three of 164 (2%) asymptomatic women (OR 18.9, 95% CI 5.73–62.7, *p* < 0.001).

**Conclusions:**

There was a significant association between PVI, as detected by transvaginal duplex imaging, and CPP. Pelvic varices were strongly associated with CPP and were infrequently seen in control patients. These results justify further evaluation of PVI and its treatment in well-designed research.

## 1 Introduction

Chronic pelvic pain (CPP) affects 26% of women worldwide.^1^ It primarily affects younger women, and is a leading cause of reduced quality of life, secondary to physical, psychological and emotional disturbances.^2^ Many women never achieve a diagnosis, and are often subjected to repeated hospital admissions and invasive investigations such as laparoscopy.^3^ Failure to reach a diagnosis that explains the symptoms is common, resulting in many women being offered hysterectomy, which itself is frequently unsuccessful.^4^

Pelvic vein incompetence (PVI) has been considered as a potential cause for CPP in women.^5,6^ Defined as retro-grade flow along the ovarian and/or internal iliac veins,^7^ PVI remains poorly understood and is often missed on laparoscopy, as the distended pelvic veins empty when patients are supine and in Trendelenburg position. Persistent retrograde flow in the pelvic venous system results in chronic venous hypertension and the development of pelvic varices around the ovaries and uterus, buttocks, vulva and lower limbs.^8,9^ Several treatments have been studied for PVI and pelvic varices, including pelvic vein ligation,^10^ coil embolisation,^11^ hormonal therapy,^12^ and hysterectomy.^13^ Despite advancement in imaging and treatment options, the association between PVI and CPP remains unclear. The prevalence of the condition in women with and without CPP is uncertain. This paucity of evidence has meant that there is no guidance on the management of PVI from the Royal College of Obstetricians and Gynaecologists (RCOG) or the UK's National Institute for Health and Care Excellence (NICE).

We sought to undertake the first high-quality, adequately powered study investigating the association between CPP and PVI or pelvic varices. We planned to determine whether the prevalence of PVI and pelvic varices in women with CPP is higher than that in age- and parity-matched women who do not have CPP.

## 2 Methods

### 2.1 Study design

Between January 2017 and February 2020, participants were enrolled into a prospective case–control study conducted at two large university teaching hospitals in north-west England. Local ethics committee approval was obtained (National Research Ethics Service ref. 13/NW/0227). All potential participants were provided with study information leaflets and written consent was obtained from those who agreed to take part. A study steering committee provided study oversight, and a diagnostic committee ensured that participants met the inclusion criteria.

### 2.2 Participants

#### 2.1.1 Cases

Premenopausal women aged 18–54 years (inclusive) with unexplained CPP were eligible for inclusion in the study. The patient's gynaecologist was required to confirm that these patients fulfilled the RCOG criteria for CPP,^14^ and that no other investigations were planned (Table 1). It was felt appropriate to recruit patients when the gynaecologist had reached the point that they would normally discharge the patient back to primary care or to a chronic pain service. We anticipated that most women would have undergone detailed investigations, including pelvic ultrasound scan and laparoscopy, but no specific investigations were mandated before a woman could be recruited.

Women were excluded if they were pregnant or within 12 months of pregnancy, found to have alternative pathologies that may cause CPP, had undergone previous hysterectomy or were unable to give informed consent.

#### 2.2.2 Controls

Premenopausal women aged between 18 and 54 years (inclusive) with no history of CPP were adopted as controls. Controls were recruited using the ‘snowball’ technique, where symptomatic women invited two asymptomatic friends to participate. Controls were also recruited through public-facing advertisements.

All participants completed a screening questionnaire to ensure that they satisfied the inclusion criteria. These and other relevant clinical information were reviewed by the study diagnostic committee. All participants were reimbursed travel expenses and received a single monetary payment for taking part in the study.

### 2.3 Transvaginal duplex ultrasound for PVI and pelvic varices

All transvaginal duplex ultrasounds (TVDUs) were performed by the same two experienced vascular scientists using a standardised protocol. Previous pilot data have demonstrated interobserver variability to be low between vascular scientists (*κ* = 0.84, very good agreement; *p* = 0.001).^15^ Both vascular scientists were blinded as to whether the participant was a case or a control. A duplex probe was introduced into the vaginal vault in the supine position and the internal iliac and ovarian veins were imaged bilaterally. Vein diameter, and directional blood flow at rest and following the Valsalva manoeuvre were recorded. The procedure was repeated with the patient in the semi-standing position.

Pelvic vein incompetence (PVI) was defined by a Delphi consensus study of British Society of Interventional Radiology (BSIR) members as ‘sustained reflux >0.7 seconds in the ovarian and/or internal iliac veins generated by Valsalva's manoeuvre’.^7^ Pelvic varices are defined as ‘the presence of tortuous, often dilated, vulval, adnexal, para-uterine veins arising from incompetent internal iliac or ovarian veins’, and were also reported.^11^ TVDU was not performed to determine any additional intra-abdominal or pelvic pathology. Previous studies and our group experience have demonstrated TVDU sensitivity and specificity of 96% and 100%, respectively, to the reference standard catheter venography.^16^

### 2.4 Outcome measures

The primary outcome measure was the presence of PVI or pelvic varices on TVDU.

Secondary outcome measures included health-related quality of life, assessed using the EuroQol (EQ-5D-3L) instrument, and pain determined by visual analogue scale (VAS).^17,18^ All subjects were asked to complete a structured questionnaire on pelvic symptoms on the day of their TVDU.

### 2.5 Sample size

The frequency of PVI in women with pelvic symptoms remains unknown. We found only four studies on the frequency of PVI in women, only one of which was sufficiently powered to use as a guide for our sample size calculation.^19^ The prevalence of asymptomatic PVI in 273 women with a mean age of 43 years was 3.7% in the left ovarian vein. Assuming that left ovarian vein incompetence represents half of the total number with PVI, the prevalence of asymptomatic PVI is likely to be around 8%. Our pilot study of 50 asymptomatic women found the frequency of PVI to be 12%.^20^ Our study group and patient advisory group deemed that the frequency of PVI in symptomatic women would need to be double that found in controls (difference of 50%) to be clinically important. The sample size calculation is based on a two-sample two-sided comparison of the prevalence of PVI by chi-square test, at the 5% significance level. The sample size was based on frequencies of 24% in the case group and 12% in the control group. To achieve 80% power in detecting a difference of 12% (i.e. 12% controls vs 24% cases), a total of 160 participants per group were required.

### 2.6 Statistical analysis

Summary statistics are presented as mean (SD) or median (range) for continuous variables, and as numbers and percentages for categorical variables. The primary analysis was a comparison of the prevalence of PVI between women with and without CPP, using the two-sided chi-square test. Logistic regression was used to compare the odds of having PVI after adjusting for a priori confounding variables of age and parity. The odds of having pelvic varices were also compared between women with and without CPP using logistic regression. The secondary analyses compared quality-of-life measures between women with CPP + PVI and women with CPP + no PVI, using the non-parametric Wilcoxon rank sum test and chi-square test, as appropriate. Published UK social preference weightings were used to transform EQ-5D-3L scores into a measure of health-related quality of life (HR-QoL).^21^ All analyses were conducted at the 5% significance level using R 4.0.4 (https://www.r-project.org).

### 2.7 Patient and public involvement

Our patient and public involvement (PPI) group includes five women with CPP and PVI, three of whom had been treated for PVI with coil embolisation. The chair of the regional charitable organisation Pelvic Pain Support Network was also a member of our PPI group, and a co-applicant during the development of this proposal. The group pilot tested our questionnaires, ensuring they were clearly understandable. The PPI group also informed the sample size calculation method and advised that the frequency of PVI in women with CPP would need to be at least double the frequency in healthy women before they could justify investigation and treatment. Members of the group were seen as active partners rather than subjects in the study. The chairperson of the group was a co-applicant in the National Institute for Health and Care Research (NIHR) funding application and a member of the study management team.

## 3 Results

**Figure 1:**
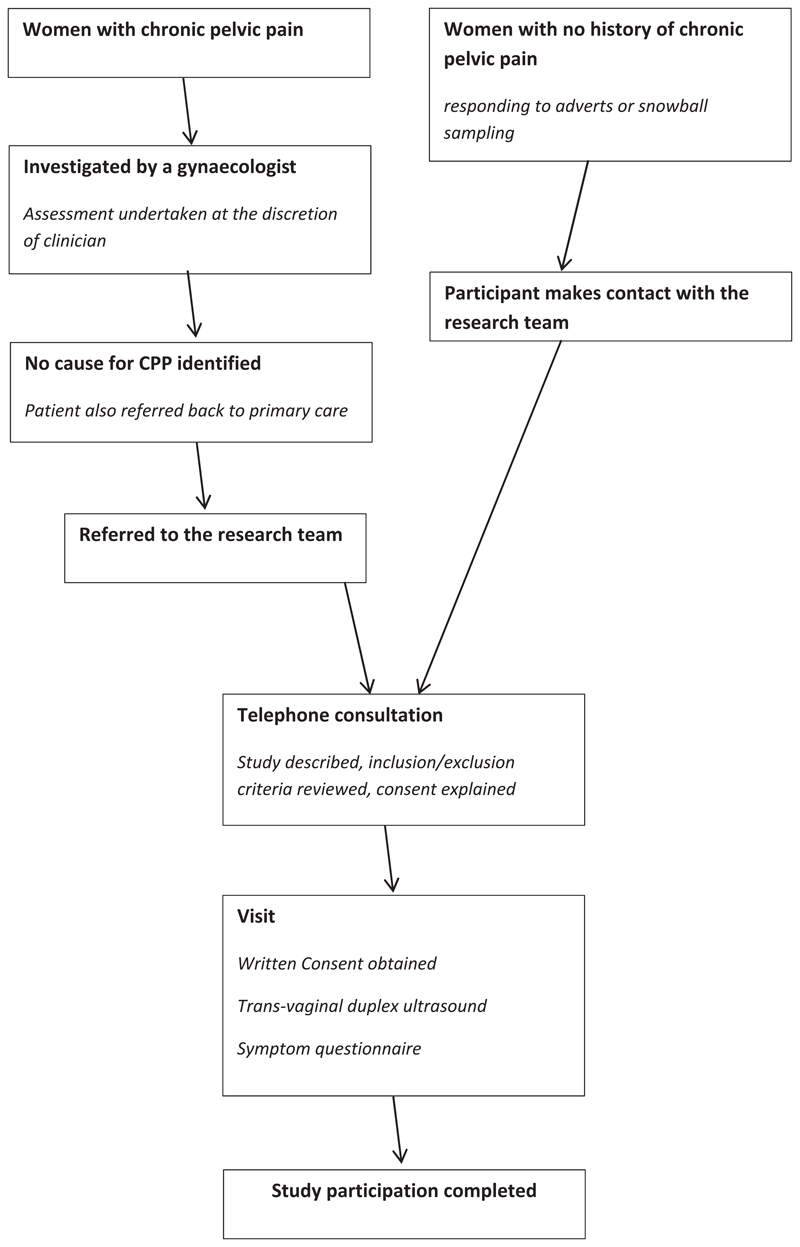
Patient flow diagram.

In total, 328 women were recruited to this study (164 cases with CPP and 164 controls without CPP). Recruitment was undertaken in north-west England at two large university teaching hospitals serving a population of 2.8 million people. Figure 1 demonstrates the recruitment and flow of participants within this study.

### 3.1 Study group characteristics

The mean (range) age was 33 years (18–53 years) for CPP cases, compared with 35 years (19–53 years) for asymptomatic controls (Table 2). CPP cases had a higher median gravidity of 2 (0–8), compared with 1 (0–6) in controls. Similarly, CPP cases had a higher median parity of 2 (0–7), compared with 1 (0–4) in controls. The body mass index was similar in both cohorts, and smoking history was more frequent in CPP cases at 22%, versus 8% in asymptomatic controls.

Patients with CPP had a significantly lower quality of life, as measured by EQ-5D-3L utility score. The median (range) score in cases was 0.76 (−0.74, 1) compared with 1 (0.41, 1) for asymptomatic controls (*p* < 0.001). The median (range) score for EQ-5D-VAS was also lower at 70 (10–100) for CPP cases, compared with 90 (50–100) for asymptomatic controls (*p* < 0.001). Pain was the primary limiting factor for quality of life in patients with CPP.

### 3.2 Primary outcome: frequency of PVI and pelvic varices

Pelvic vein incompetence diagnosed on duplex ultrasound was significantly more frequent in women with CPP, at 101/164 (62%) compared with 31/164 (19%) in asymptomatic controls (unadjusted OR 6.79, 95% CI 4.11–11.47, *p* < 0.001). This difference remained equally significant after adjusting for age and parity (adjusted OR 6.02, 95% CI 3.52–10.56, p < 0.001).

The overall frequency of pelvic varices was lower, found in 43 of 164 (26%) women with CPP compared with only three of 164 (2%) controls without CPP (OR 18.9, 95% CI 5.73–62.7, *p* < 0.001).

### 3.3 Secondary outcomes

When comparing health status, there was no significant difference in EQ5D utility score between women with PVI and CPP compared with women with CPP only, with median (range) values of 0.73 (−0.08, 1) with PVI and 0.76 (−0.7, 1) without PVI (*p* = 0.16).

### 3.4 Symptoms associated with PVI

Women with PVI more commonly described their pain as ‘throbbing’ (53% vs 29%, *p* < 0.001) and ‘aching’ (77% vs 65%, *p* = 0.04). Other descriptors shared equally with women without PVI were ‘dull’, ‘spasmodic’, ‘sharp’ and ‘cramping’ (Table 3). Women with PVI and CPP reported more regular periods (60% vs 41%, *p* = 0.02) and less intermenstrual bleeding (35% vs 60%, *p* = 0.002). Pain before and during the menstrual cycle was reported equally in both groups. No significant difference was seen in symptoms of abdominal bloating, lower back pain or pain on defecation.

## 4 Discussion

### 4.1 Main findings

This prospective case–control study demonstrates that PVI was strongly associated with CPP when compared with matched asymptomatic controls. More significantly, the presence of pelvic varices has an even stronger association with CPP. The study has also demonstrated that women with PVI have a distinctive symptom profile that may help clinicians reach an earlier diagnosis. The most notable features include the presence of non-cyclical ‘dull’ and ‘throbbing’ pelvic pain that radiates to the upper thighs and is exacerbated by sexual intercourse. Women with PVI have regular periods and rarely experience intermenstrual bleeding. A large proportion of women with PVI had previous pregnancies, with only 19% of women with PVI and CPP being nulliparous. Only one patient with pelvic varices was nulliparous, which does suggest that pregnancy may be a risk factor for the development of PVI. We hypothesise that the massive increase in circulating blood volume and the presence of a growing gravid uterus leads to substantial increases in pelvic venous flow, which can result in vessel diameter expansion to the point where valves fail, precipitating reflux. These changes can persist after pregnancy.^22^

This study has shown PVI to be present on TVDU in 19% of asymptomatic, healthy women. It is therefore likely that the presence of PVI is not necessarily pathological in isolation, but the presence of pelvic varices does demonstrate a pathological transformation possibly as a result of exaggerated venous stasis. Pelvic varices were rarely seen in asymptomatic women, and the strong association between pelvic varices and CPP would suggest that it is these women that may benefit from further investigation and treatment in the form of catheter venography and coil embolisation, although the effectiveness of this intervention has not been demonstrated in randomised controlled trials.

### 4.2 Strengths and limitations

This present study was strengthened by prospective recruitment, the matching of case–control pairs and no loss to follow-up. This study used predefined definitions for PVI, pelvic varices and CPP, which were determined by Delphi consensus.^12^ All cases were thoroughly evaluated before participating in the study, and controls were invited to complete a screening questionnaire to ensure that the inclusion criteria were met. Participants underwent a standardised TVDU protocol performed by one of two trained vascular technologists. TVDU has grown in popularity as a non-invasive, safe alternative to reflux venography. It is a dynamic test that allows the venous system to be integrated with the Valsalva manoeuvre in the supine and in the semi-standing position. Previous studies have demonstrated sensitivity and specificity of 96% and 100%, respectively, against the reference standard catheter venography.^16^ Inter-observer variability has also been shown to be high (*κ* = 0.84; *p* = 0.001).^22^ As both cases and controls were evaluated for PVI using the same TVDU protocol, the comparison of PVI frequency between the groups can be assumed to be correct. Previous research on PVI has being flawed with heterogenous definitions of PVI and CPP, and a lack of strict inclusion criteria.^23^

Limitations include the possibility of control patients having an underlying gynaecological or pelvic condition. All cases were evaluated by a gynaecologist and were investigated, although no minimal requirement was established. Control patients did not undergo any formal investigations to determine the presence or absence of conditions such as adenomyosis, endometriosis or similar. Although this may impact the results of the symptom questionnaire individually, it is unlikely to have impacted the results of the trans-vaginal duplex ultrasound findings. Information on the patient's hormone profile and menstrual cycle was not collected.

The recruitment target for this study was 160 patients per group. During the study design, this target was extrapolated to allow for a 10% attrition rate (176 patients per group). However, given that there was no loss to follow-up, the recruitment target was subsequently reduced to the minimum required.

### 4.3 Interpretation

Prior to this study, the association between PVI and CPP had not been rigorously investigated, nor had the prevalence of PVI in women with and without symptoms been identified. The current evidence base includes a number of small, inadequately powered studies with participant numbers ranging from 22 to 151. Many studies diagnosed PVI as an incidental finding, such as women undergoing computed tomography (CT) for the follow-up of metastatic disease.^24^ A larger retrospective study of 273 CT scans of female patients in a renal donor programme identified left ovarian vein incompetence in 9.9%.^19^ The right ovarian and internal iliac veins were not assessed. Interestingly, half of those patients found to have left ovarian vein incompetence also had CPP. Other studies selectively recruited patients with lower limb venous insufficiency that would not represent the general female population given the strong correlation between PVI and lower limb venous incompetence.^25,26^ A smaller study of 100 women with CPP of indeterminate aetiology were investigated using both TVDU and lower limb Doppler ultrasound. PVI was identified in 30 of 100 (30%) women. Of those 30 women, 21 (70%) were found to have associated lower limb venous insufficiency.^27^

Chronic pelvic pain is a significant health burden, and the list of differential diagnoses is vast. Women are frequently subjected to multiple investigations and invasive procedures to identify a cause for their symptoms. Clinicians never reach a diagnosis in approximately 50% of women who present with symptoms of CPP, and so it is essential that alternative causes of CPP are explored. The results of this study provide justification for PVI to be investigated as a cause for CPP and for treatment efficacy to be evaluated in well-designed randomised control trials.

This study highlights the need for patients with CPP to be assessed using a multidisciplinary approach with PVI being considered as a diagnosis with early referral for investigation. This may reduce the need for diagnostic laparoscopy and even hysterectomy. Coil occlusion has been evaluated in our group's randomised controlled trial. Should emergent trial data demonstrate a beneficial effect of treatment of PVI for CPP, this would influence clinical guidelines and the commissioning policy to develop treatment pathways for the investigation and treatment of PVI. Endovascular interventions already exist to treat PVI, and under these circumstances, research results demonstrating the role of PVI in the causation of CPP could quickly be incorporated into clinical services.

Future research should ensure that patients’ symptoms and radiological findings are appropriately defined, using acceptable classifications,^9^ and should include the use of reliable outcome measures. The strong association between CPP and pelvic varices would benefit from further exploration in the form of a randomised control trial of intervention, with long-term follow-up assessing reoccurrence rates and long-term changes in venous morphology.

## 5 Conclusion

This study has found a highly significant association between CPP and PVI. Notably, there was an even stronger association between pelvic varices and CPP. Our study identifies that women with PVI have a distinctive symptom profile. Pain was commonly described as ‘dull’ and/or ‘throbbing’, unrelated to the menstrual cycle or sexual intercourse. This study justifies urgent research into PVI and its treatment.

## Figures and Tables

**Table 1:** Inclusion and exclusion criteria.

	Inclusion criteria	Exclusion criteria
Case	18–54 years of age Premenopausal Chronic pelvic pain^a^ All causes for pelvic pain investigated by a gynaecologist	Pregnant (or within 12 months or pregnancy) Identified to have, or a history of, pathology associated with chronic pelvic pain Previous hysterectomy Unable to provide informed consent
Control	18–54 years of age Premenopausal Nohistory of chronic pelvic pain	Past or current history of chronic pelvic pain Pregnant (or within 12 months or pregnancy) Identified to have, or a history of, pathology associated with chronic pelvic pain Previous hysterectomy Unable to provide informed consent

^a^Chronic pelvic pain (CPP) is defined as intermittent or constant pain in the lower abdomen or pelvis of a woman of at least 6 months in duration, not occurring exclusively with menstruation or intercourse and not associated with pregnancy.^14^

**Table 2:** Cohort characteristics.

	Women with CPP (*n* = 164)	Asymptomatic controls (*n* = 164)
Mean age (range)	33 (18–53)	35 (19–53)
Median gravida (range)	2 (0–8)	1 (0–6)
Median parity (range)	2 (0–7)	1 (0–4)
Mean BMI (range)	25 (14–42)	25 (18–46)
Current smoker (%)	36 (22)	13 (8)

**Table 3:** Pain descriptors, chronology and associated features in women with pelvic vein incompetence (PVI) and chronic pelvic pain (CPP), versus women with CPP only.

	Women with CPP and PVI (*n* = 101)		Women with CPP only (*n* = 63)		
	*f* (%)		*f* (%)		*p*
Descriptor					
Dull	62 (61)		35 (56)		0.6
Throbbing	63 (62)		35 (29)		<0.001
Spasm	24 (24)		31 (25)		0.3
Ache	91 (90)		50 (80)		0.04
Shooting	41 (41)		34 (28)		0.4
Burning	34 (34)		14 (11)		0.002
Sharp	50 (50)		50 (41)		0.2
Cramp	58 (57)		45 (71)		0.3
Chronology and associated features					
Regular periods	59 (58)		24 (38)		0.02
Pain before periods	91 (90)		57 (90)		0.82
Pain during periods	90 (89)		55 (87)		0.29
Intermenstrual bleeding	33 (33)		35 (56)		0.002
Dyspareunia	65 (64)		42 (63)		0.19
Pain on defecation	41 (41)		28 (44)		0.4
Lower back pain	86 (85)		53 (84)		0.4

## Data Availability

The data that support the findings of this study are available on request from the corresponding author. The data are not publicly available due to privacy or ethical restrictions.

## References

[1] Daniels JP, Khan KS (2010). Chronic pelvic pain in women. BMJ.

[2] McGowan L, Luker K, Creed F, Chew-Graham CA (2007). How do you explain a pain that can't be seen?: the narratives of women with chronic pelvic pain and their disengagement with the diagnostic cycle. Br J Health Psychol.

[3] Lamvu G, Carrillo J, Ouyang C, Rapkin A (2021). Chronic pelvic pain in women: a review. JAMA.

[4] Cockrum R, Tu F (2022). Hysterectomy for chronic pelvic pain. Obstet Gynecol Clin North Am.

[5] Tu FF, Hahn D, Steege JF (2010). Pelvic congestion syndrome-associated pelvic pain: a systematic review of diagnosis and management. Obstet Gynecol Surv.

[6] Champaneria R, Shah L, Moss J, Gupta J, Birch J, Middleton L (2016). The relationship between pelvic vein incompetence and chronic pelvic pain in women: systematic reviews of diagnosis and treatment effectiveness. Health Technol Assess.

[7] Riding D, Pond E, McCollum CN, Caress AL (2019). Seeking consensus amongst UK-based interventional radiologists on the imaging diagnosis of pelvic vein incompetence in women with chronic pelvic pain: a modified Delphi study. Phlebology.

[8] Phillips D, Deipolyi AR, Hesketh RL, Midia M, Oklu R (2014). Pelvic congestion syndrome: etiology of pain, diagnosis, and clinical management. J Vasc Interv Radiol.

[9] Meissner MH, Khilnani NM, Labropoulos N, Gasparis AP, Gibson K, Greiner M (2021). The symptoms-varices-pathophysiology classification of pelvic venous disorders: a report of the American Vein < Lymphatic Society International Working Group on pelvic venous disorders. J Vasc Surg Venous Lymphat Disord.

[10] Gargiulo T, Mais V, Brokaj L, Cossu E, Melis GB (2003). Bilateral laparoscopic transperitoneal ligation of ovarian veins for treatment of pelvic congestion syndrome. J Am Assoc Gynecol Laparosc.

[11] Lopez AJ (2015). Female pelvic vein embolization: indications, techniques, and outcomes. Cardiovasc Intervent Radiol.

[12] Soysal ME, Soysal S, Vicdan K, Ozer S (2001). A randomized controlled trial of goserelin and medroxyprogesterone acetate in the treatment of pelvic congestion. Hum Reprod.

[13] Chung MH, Huh CY (2003). Comparison of treatments for pelvic congestion syndrome. Tohoku J Exp Med.

[14] Moore SJ, Kennedy SH (2012). Royal College of obstetrics and Gynaecology green-top guideline: the initial management of chronic pelvic pain.

[15] Hansrani V, Dhorat Z, McCollum CN (2017). Diagnosing of pelvic vein incompetence using minimally invasive ultrasound techniques. Vascular.

[16] Barros FS, Perez JMG, Zandonade E, Salles-Cunha SX, Monedero JL, Hilel ABS (2010). Evaluation of pelvic varicose veins using color doppler ultrasound: comparison of results obtained with ultrasound of the lower limbs, transvaginal ultrasound, and phlebography. J Vasc Bras.

[17] EQ-5D 3 Level.

[18] Dolan P (1997). Modeling valuations for EuroQol health states. Med Care.

[19] Belenky A, Bartal G, Atar E, Cohen M, Bachar GN (2002). Ovarian varices in healthy female kidney donors: incidence, morbidity, and clinical outcome. AJR Am J Roentgenol.

[20] Riding DM, Hansrani V, Norse K, Morris J, Seif M, McCollum C (2017). The frequency of pelvic vein incompetence in women with chronic pelvic pain: an interim report. BJOG.

[21] Szende AOM, Devlin N (2006). EQ-5D value sets: inventory, comparative review and user guide.

[22] Palmer SK, Zamudio S, Coffin C, Parker S, Stamm E, Moore LG (1992). Quantitative estimation of human uterine artery blood flow and pelvic blood flow redistribution in pregnancy. Obstet Gynecol.

[23] Khilnani NM, Meissner MH, Learman LA, Gibson KD, Daniels JP, Winokur RS (2019). Research priorities in pelvic venous disorders in women: Recommendations from a Multidisciplinary Research Consensus Panel. J Vasc Interv Radiol.

[24] Hiromura T, Nishioka T, Nishioka S, Ikeda H, Tomita K (2004). Reflux in the left ovarian vein: analysis of MDCT findings in asymptomatic women. Am J Roentgenol.

[25] Asciutto G, Asciutto KC, Mumme A, Geier B (2009). Pelvic venous incompetence: reflux patterns and treatment results. Eur J Vasc Endovasc Surg.

[26] Perrin MR, Labropoulos N, Leon LR (2006). Presentation of the patient with recurrent varices after surgery (REVAS). J Vasc Surg.

[27] Gultasli NZ, Kurt A, Ipek A, Gumus M, Yazicioglu KR, Dilmen G (2006). The relation between pelvic varicose veins, chronic pelvic pain and lower extremity venous insufficiency in women. Diagn Interv Radiol.

